# The Story of the Insane from Year to Year

**Published:** 1897-06-12

**Authors:** 


### THE STORY OF THE INSANE FROM YEAR TO YEAR.

Tenth Series.

### BARMING HEATH ASYLUM, KENT.

On January 1st, 1896, there were 1,606 patients in this asylum, and on the last day of the year the number had risen to 1,661. There were 284 admissions, 40 readmissions, 107 discharged recovered, 15 discharged relieved, and 138 deaths. The percentage of recoveries on admissions was 32·9, and that of the deaths on the average number resident was 8·7. Of the deaths 42 were caused by cerebral diseases, 34 by consumption, and 10 by pneumonia. Of the year's admissions 37 owed their insanity to various moral causes, such as domestic trouble, religion, and love; 13 to intemperance in drink; and 84 to hereditary tendency; while in 35 the cause was not ascertained. Dr. Davies states that the asylum has been entirely free from infectious disease, and that there has been no suicide, no mechanical restraint, and no seclusion. Lectures are given to the attendants and nurses, and 11 have passed the Medico-Psychological Examination. The visitors have wisely granted an increase of wages to all those attendants who hold the certificate. The asylum is somewhat crowded, and there does not seem much hope of relieving this until the additions to the Chartham Asylum are opened. The profits on the farm are stated to be £2,738, but, on looking over the farm accounts, we see nothing charged for rent or interest on capital, or for patients' labour; and no prices are given of the articles sent from the farm to the asylum. The weekly cost per head is 9s.

### THE COUNTY ASYLUM, LEICESTER.

During the year 1896 the number increased from 462 to 505. There were 93 admissions, 42 readmissions, 82 recoveries, 8 discharged relieved, and 50 deaths. The recoveries stand at 23·7 per cent. on the admissions, and the deaths at 10 per cent. on the average numbers resident; the former is below the county asylum average and the latter is just about the average. Twenty of the deaths were caused by cerebral and spinal diseases, 11 by consumption, 3 by pneumonia, and 1 by enteritis. Of the admissions, 19 owed their insanity to moral causes, 17 to drink, and 39 to hereditary tendency. Two new blocks for 50 patients each were formally opened on May 12th, and were filled before the end of the year. This asylum is an old one, and it was proposed to make various alterations, and plans for these were prepared, but the Lunacy Commissioners refused to sanction them. The visitors reconsidered the matter, and finally decided that further additions were not advisable, and they recommended that a new asylum be built on a fresh site. This was proposed many years ago, but for some reason or other it fell through. This asylum has a charitable fund attached to it, and 30 patients availed themselves of this fund during the year. We are pleased to be able to congratulate Attendant Thomas Norman on having obtained his pension of £45 per annum, and we also congratulate Samuel Burdett on having obtained his pension of £54 12s. per annum. The farm is credited with a balance in its favour of £357, but again we see no evidence that rent, interest on capital, or patients' labour has been reckoned, and the prices at which articles are supplied to the house are not given, so very little can be learned from the balance-sheet. The weekly cost per head is 8s. 5¼d.

### THE CORK DISTRICT ASYLUM.

The limits of accommodation are given at 1,200, but there were 1,308 in residence at the close of the year. There were 236 admissions, 55 readmissions, 91 discharged recovered, 11 discharged relieved, and 84 deaths. The recoveries stand at 31·3 per cent. of the admissions, and the deaths at 6·4 per cent. of the average number resident. Cerebral and spinal diseases were the causes of death in 18 cases, consumption in 36, dysentery in 1, and there was one case of suicide. In 38 of the admissions moral causes are credited with the cause of the insanity, intemperance in drink in 39, hereditary influence in 94, and previous attacks in 35. Dr. Woods says that 50 per cent. of the admissions were incurables, and it is probable that many of these would have recovered if they had been put under treatment at an early period of their illness. When referring to their high percentage of heredity, Dr. Woods asks, Why should not the law deal with this social blot? And we echo, Why not? At the same time we have scarcely a particle of hope that anything will be done. In the State of Connecticut it has been made a felony for any epileptic, imbecile, or weak-minded man or woman to marry; but then they manage these things better in America, and it will be a long time before a similar law finds a place in the English Statute Book. There were four cases of mild typhoid fever during the spring. They arose in different parts of the house, and were not attributed to sanitary defect. This has been often noted elsewhere, and it would seem certain that either typhoid, or a disease almost identical, does occasionally make its appearance when it is impossible to trace its origin either to bad sanitary arrangement, impure water, or contaminated milk. It is proposed to build a hospital for the treatment of acute cases, and Dr. Woods thinks, and thinks rightly, that paying patients could be received at remunerative charges, but if he also thinks that he will increase his percentage of recoveries we fear he will be mistaken. The average annual cost per head was £21 15s. 7d.

